# Sterilizing activity of spectinamide MBX-4888A when replacing linezolid in the Nix-TB regimen in the relapsing BALB/c mouse model of tuberculosis

**DOI:** 10.1101/2025.08.04.668403

**Published:** 2025-08-06

**Authors:** Nathan Peroutka-Bigus, Michael S. Sherman, Firat Kaya, Samanthi L. Waidyarachchi, Jiuyu Liu, Joel Rushefsky, Michelle M. Butler, Terry Bowlin, Bernd Meibohm, Mercedes Gonzalez-Juarrero, Anne J. Lenaerts, Matthew Zimmerman, Richard E. Lee, Gregory T. Robertson

**Affiliations:** a Mycobacteria Research Laboratories, Department of Microbiology, Immunology and Pathology, Colorado State University, Fort Collins, Colorado, USA.; b Center for Discovery and Innovation, Hackensack Meridian School of Medicine, Nutley, New Jersey, USA.; c Microbiotix, Inc., Worcester, MA 01605, USA; d Department of Chemical Biology & Therapeutics, St. Jude Children’s Research Hospital, Memphis, Tennessee, USA; e Department of Pharmaceutical Sciences, University of Tennessee Health Science Center, Memphis, TN 38163, USA.

**Keywords:** Tuberculosis, relapse, BALB/c, spectinamide, bedaquiline, pretomanid

## Abstract

Spectinamides have garnered interest as experimental tuberculosis therapeutics owing to their safety profile and efficacy as partner agents when used in conjunction with established regimens in mice. The Nix-TB regimen of bedaquiline, pretomanid, and linezolid represents a short, effective regimen recommended for treatment of pre-extensively drug-resistant tuberculosis. However, linezolid administration is associated with severe adverse events which limits its use. Here we present preclinical data that spectinamide MBX-4888A can replace linezolid in Nix-TB.

The effective treatment of patients with all forms of tuberculosis (TB) is critical in the fight to eliminate the spread of drug-resistant *Mycobacterium tuberculosis* (*Mtb*). Combination antibiotic regimens intended for the treatment of patients with drug-resistant TB are associated with severe adverse events (AEs), which complicates treatment, leading to treatment discontinuation, non-compliance, and further expansion of drug resistance (1). The Nix-TB Phase 3 clinical trial demonstrated exceptional cure rates in patients with drug-resistant forms of TB using a three-drug regimen consisting of bedaquiline (B), pretomanid (Pa), and linezolid (L) (2). While the BPaL regimen is efficacious in treating drug resistant forms of TB, long-term administration of L was associated with AEs of peripheral neuropathy, myelosuppression, optic neuritis, and anemia, leading to dose reductions and frequent interruptions in L treatment (2). Spectinamide MBX-4888A (4888A) represents a new class of semisynthetic antibiotics that shows promising activity against *Mtb* in diverse preclinical murine models of TB disease with favorable safety profiles (3–9). Like L, 4888A targets protein synthesis, but occupies a different binding site in the ribosome (5).

Spectinamides are promising partner agents when administered with standard combination TB treatment regimens. The inclusion of spectinamide Lee-1599 with BPa resulted in increased bactericidal activity in a high dose aerosol subacute TB BALB/c infection model (4); while 4888A, given by injection with the front line standard of care regimen, led to improved bactericidal responses and treatment shortening in the subacute relapsing BALB/c model and in the more rigorous spectrum of disease chronic C3HeB/FeJ relapsing model of advanced pulmonary disease (8). An inhaled formulation of spectinamide Lee-1599 showed similar efficacy, but with reduced toxicities and an absence of known hematological effects, when used as a replacement for L in the BPaL regimen in BALB/c and C3HeB/FeJ mice (9). However, those studies failed to address whether a spectinamide when administered in place of L in BPaL resulted in durable sterilizing cure as measured by the prevention of relapse after treatment completion. This study employed the high-dose aerosol BALB/c relapsing mouse model to evaluate whether the injectable compound 4888A could replace L in the BPaL regimen, while still achieving a durable cure within a similarly short treatment duration.

One day following high dose aerosol with *Mtb* Erdman (see Methods in supplemental materials), average CFU lung burdens in female BALB/c mice were 4.04 log10 CFU and increased to 7.42 log10 CFU ten days later at the start of treatment. During treatment, both BPaL and BPa4888A significantly reduced *Mtb* lung burdens. With 4-weeks of therapy (5 of 7 days per week) BPa4888A reduced *Mtb* lung burdens by 4.98 log10 CFU, which was not significantly different from the 4.91 log10 CFU reduction with BPaL therapy (Fig 1A, Table S1). Following 8-weeks of therapy both treatments reduced *Mtb* lung burdens to below the limit of detection (> 6.54 log10 CFU), suggesting the presumptive clearance of *Mtb* from the lungs (Fig. 1A, Table S1). Weight loss is associated with severity of disease in this model. Following an initial period of weight decline, mice in both treatment arms gained weight throughout the remainder of the study (Fig. S1), indicating clinical improvements with treatment. Fewer relapse events were observed in mice held for 3 months after receiving 8-weeks of BPaL compared to those receiving 8-weeks of BPa4888A (29% vs 87% [P < 0.05]), with all mice achieving durable cure with 12-weeks of treatment (BPaL and BPa4888A) (Fig. 1B). Pharmacokinetic analysis based on sparse plasma sampling in infected (not shown) and healthy mice (Fig. S2) confirmed no drug-drug interactions with equivalent drug exposures for BPa and 4888A in plasma (Table S2a-d).

Although effective, clinical toxicities of L necessitate dose reduction or dosing holidays (2) which poses a serious threat to further expansion of clinical resistance to B (10). Modifications to BPaL, as the current WHO recommended pre-XDR therapy (11), are desirable to improve clinical control of TB. Although restricted currently to injection or inhaled therapies, spectinamides exhibit favorable safety profiles and tolerability in preclinical studies (3, 8, 9), and as shown here, appear capable of contributing to durable cure in the BALB/c relapsing mouse model as a replacement for L in the BPaL regimen. Indeed, in a previous study, 4888A was administered subcutaneously for 20 weeks in *Mtb*-infected mice, providing an additional data point regarding the safety and tolerability of spectinamide 4888A (8). Our studies further the available data on the utility of spectinamides in the treatment of TB. While these results are presently limited to outcomes in the conventional BALB/c relapsing mouse model - thought to best represent uncomplicated TB disease - our prior work demonstrated effective distribution and accumulation of 4888A within the caseous lesions of C3HeB/FeJ mice (8), suggesting a possible role for BPa4888A in harder to treat patients with necrotic acellular caseum filled granulomas; this remains to be tested.

This study has several limitations. BPa was not tested as a direct comparator, so the contribution of 4888A to treatment efficacy and cure cannot be estimated. However, previous work showed that a closely related spectinamide, Lee-1599, does add appreciably to the BPa backbone in this same infection model (4). Further studies are needed to de-convolute the contribution of 4888A to cure in this preclinical model. Additionally, while sparse PK assessment provided data that co-administration of 4888A with BPa did not result in any drug-drug interactions, a full PK analysis was not performed, and the comparative behaviors of L and 4888A in this context were not evaluated.

The fact that both BPaL and BPa4888A achieved a 100% cure rate after 12 weeks of treatment, and that 4888A was well tolerated, adds further evidence supporting the safety and efficacy of this antibiotic. Given its unique binding site and absence of current clinical use, it is anticipated that 4888A will not be impacted by any pre-existing clinical resistance – which is not the case for linezolid (a repurposed drug) for which clinical resistance is on the rise (12, 13). One counterpoint was a significant early advantage of BPaL in reducing the number of mice that relapsed at the 8-week timepoint. As both combination regimens were anchored by BPa, one possible explanation is the nearly 100% oral bioavailability of L in humans and mice (14, 15) improves early target attainment and drives cure in drug-tolerant populations that arise during drug treatment, but other possibilities such as absolute target potency and the differences in L (16) and 4888A (17) model-based exposure responses should also be considered.

## Figures and Tables

**Figure 1.**
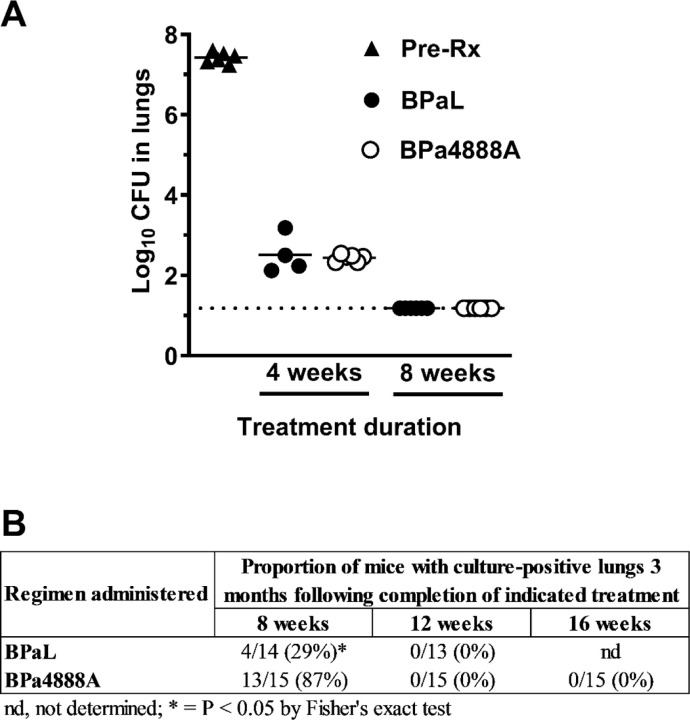
Antimicrobial regimen activity against *Mycobacterium tuberculosis* Erdman strain in BALB/c relapsing mouse model following high dose aerosol infection. **A.** Bactericidal activity of the two regimens over the 8-week treatment course. Pre-treatment (Pre-Rx) CFU lung burdens (closed triangles), 4- or 8-weeks of BPaL (closed circles), or 4- or 8-weeks of BPa4888A (open circles). **B.** The number of mice that relapsed 3 months after the indicated treatment duration over the group total. The percentage of mice relapsing is listed in parentheses. Drug doses in mg/kg: (B) Bedaquiline (25), (Pa) pretomanid (100), (L) linezolid (100), (4888A) MBX-4888A (200).

## References

[1] Udwadia Z, Furin J. 2019. Quality of drug-resistant tuberculosis care: Gaps and solutions. J Clin Tuberc Other Mycobact Dis 16:100101. 31720427 10.1016/j.jctube.2019.100101 PMC6830144

[2] Conradie F, Diacon AH, Ngubane N, Howell P, Everitt D, Crook AM, Mendel CM, Egizi E, Moreira J, Timm J, McHugh TD, Wills GH, Bateson A, Hunt R, Van Niekerk C, Li M, Olugbosi M, Spigelman M. 2020. Treatment of Highly Drug-Resistant Pulmonary Tuberculosis. N Engl J Med 382:893–902. 32130813 10.1056/NEJMoa1901814 PMC6955640

[3] Lee RE, Hurdle JG, Liu J, Bruhn DF, Matt T, Scherman MS, Vaddady PK, Zheng Z, Qi J, Akbergenov R, Das S, Madhura DB, Rathi C, Trivedi A, Villellas C, Lee RB, Rakesh, Waidyarachchi SL, Sun D, McNeil MR, Ainsa JA, Boshoff HI, Gonzalez-Juarrero M, Meibohm B, Böttger EC, Lenaerts AJ. 2014. Spectinamides: a new class of semisynthetic antituberculosis agents that overcome native drug efflux. Nat Med 20:152–158. 24464186 10.1038/nm.3458 PMC3972818

[4] Robertson GT, Scherman MS, Bruhn DF, Liu J, Hastings C, McNeil MR, Butler MM, Bowlin TL, Lee RB, Lee RE, Lenaerts AJ. 2017. Spectinamides are effective partner agents for the treatment of tuberculosis in multiple mouse infection models. J Antimicrob Chemother 72:770–777. 27999020 10.1093/jac/dkw467 PMC5400088

[5] Liu J, Bruhn DF, Lee RB, Zheng Z, Janusic T, Scherbakov D, Scherman MS, Boshoff HI, Das S, Rakesh, Waidyarachchi SL, Brewer TA, Gracia B, Yang L, Bollinger J, Robertson GT, Meibohm B, Lenaerts AJ, Ainsa J, Böttger EC, Lee RE. 2017. Structure-Activity Relationships of Spectinamide Antituberculosis Agents: A Dissection of Ribosomal Inhibition and Native Efflux Avoidance Contributions. ACS Infect Dis 3:72–88. 28081607 10.1021/acsinfecdis.6b00158 PMC5493200

[6] Gonzalez-Juarrero M, Lukka PB, Wagh S, Walz A, Arab J, Pearce C, Ali Z, Ryman JT, Parmar K, Temrikar Z, Munoz-Gutierrez J, Robertson GT, Liu J, Lenaerts AJ, Daley C, Lee RE, Braunstein M, Hickey AJ, Meibohm B. 2021. Preclinical Evaluation of Inhalational Spectinamide-1599 Therapy against Tuberculosis. ACS Infect Dis 7:2850–2863. 34546724 10.1021/acsinfecdis.1c00213 PMC10226119

[7] Liu J, Lukka PB, Ektnitphong VA, Parmar KR, Wagh S, Lu Y, Lee RB, Scherbakov D, Wang H, Zimmerman MD, Meibohm B, Robertson GT, Dartois V, Böttger EC, Lenaerts AJ, Lee RE. 2024. Enhancing the therapeutic window for Spectinamide anti-tuberculosis Agents: Synthesis, Evaluation, and activation of phosphate prodrug 3408. Bioorg Med Chem Lett 112:129934. 39214506 10.1016/j.bmcl.2024.129934 PMC11403708

[8] Bauman AA, Sarathy JP, Kaya F, Massoudi LM, Scherman MS, Hastings C, Liu J, Xie M, Brooks EJ, Ramey ME, Jones IL, Benedict ND, Maclaughlin MR, Miller-Dawson JA, Waidyarachchi SL, Butler MM, Bowlin TL, Zimmerman MD, Lenaerts AJ, Meibohm B, Gonzalez-Juarrero M, Lyons MA, Dartois V, Lee RE, Robertson GT. 2024. Spectinamide MBX-4888A exhibits favorable lesion and tissue distribution and promotes treatment shortening in advanced murine models of tuberculosis. Antimicrob Agents Chemother 68:e0071624. 39345140 10.1128/aac.00716-24 PMC11539231

[9] Zohaib Ali M, Dutt TS, MacNeill A, Walz A, Pearce C, Lam H, Philp JS, Patterson J, Henao-Tamayo M, Lee R, Liu J, Robertson GT, Hickey AJ, Meibohm B, Gonzalez Juarrero M. 2024. A modified BPaL regimen for tuberculosis treatment replaces linezolid with inhaled spectinamides. Elife 13. 10.7554/eLife.96190 PMC11460978 39378165

[10] Nguyen TVA, Anthony RM, Bañuls AL, Nguyen TVA, Vu DH, Alffenaar JC. 2018. Bedaquiline Resistance: Its Emergence, Mechanism, and Prevention. Clin Infect Dis 66:1625–1630. 29126225 10.1093/cid/cix992

[11] Global tuberculosis report 2024. Geneva: World Health Organization, 2024. Licence: CC BY-NC-SA 3.0 IGO.

[12] Nambiar R, Tornheim JA, Diricks M, De Bruyne K, Sadani M, Shetty A, Rodrigues C. 2021. Linezolid resistance in Mycobacterium tuberculosis isolates at a tertiary care centre in Mumbai, India. Indian J Med Res 154:85–89. 34782533 10.4103/ijmr.IJMR_1168_19 PMC8715694

[13] Azimi T, Khoshnood S, Asadi A, Heidary M, Mahmoudi H, Kaviar VH, Hallajzadeh M, Nasiri MJ. 2022. Linezolid resistance in multidrug-resistant Mycobacterium tuberculosis: A systematic review and meta-analysis. Front Pharmacol 13:955050. 36110536 10.3389/fphar.2022.955050 PMC9468755

[14] Stalker DJ, Jungbluth GL. 2003. Clinical pharmacokinetics of linezolid, a novel oxazolidinone antibacterial. Clin Pharmacokinet 42:1129–40. 14531724 10.2165/00003088-200342130-00004

[15] Welshman IR, Sisson TA, Jungbluth GL, Stalker DJ, Hopkins NK. 2001. Linezolid absolute bioavailability and the effect of food on oral bioavailability. Biopharm Drug Dispos 22:91–7. 11745911 10.1002/bdd.255

[16] Zhang H, He Y, Davies Forsman L, Paues J, Werngren J, Niward K, Schön T, Bruchfeld J, Alffenaar JW, Hu Y. 2022. Population pharmacokinetics and dose evaluations of linezolid in the treatment of multidrug-resistant tuberculosis. Front Pharmacol 13:1032674. 36699070 10.3389/fphar.2022.1032674 PMC9868619

[17] Wagh S, Rathi C, Lukka PB, Parmar K, Temrikar Z, Liu J, Scherman MS, Lee RE, Robertson GT, Lenaerts AJ, Meibohm B. 2021. Model-Based Exposure-Response Assessment for Spectinamide 1810 in a Mouse Model of Tuberculosis. Antimicrob Agents Chemother 65:e0174420. 34424046 10.1128/AAC.01744-20 PMC8522768

